# Shungite (Mineralized Carbon) as a Promising Electrode Material for Electroanalysis

**DOI:** 10.3390/ma16031217

**Published:** 2023-01-31

**Authors:** Milan Sýs, Michaela Bártová, Martin Bartoš, Ivan Švancara, Tomáš Mikysek

**Affiliations:** Department of Analytical Chemistry, Faculty of Chemical Technology, University of Pardubice, Studentská 573, 532 10 Pardubice, Czech Republic

**Keywords:** shungite, microscopic imaging, ferrocyanide/ferricyanide redox couple, electrochemical kinetics, electroanalytical applicability

## Abstract

In this study, two different types of amorphous carbonaceous Precambrian rock, classified as noble elite shungite and black raw shungite, were tested as possible electrode materials of natural origin. Both types were machined into cylindrical shapes to form the corresponding solid electrodes and their physicochemical and electrochemical properties were compared with the standard glassy carbon electrode (GCE). The raw stones were first subjected to microscopic imaging by using scanning electron microscopy and energy-dispersive X-ray spectroscopy, both of which indicated significant differences in their morphology and in the content of impurities. An electrode prototype manufactured from noble elite shungite (EShE) with a carbon content of about 94% (*w/w*) has offered a very satisfactory electrochemical performance with a nearly identical heterogeneous electron-transfer rate constant of 7.8 × 10^−3^ cm s^−1^ for ferro/ferricyanide redox couple, a slightly narrower potential range (~2.1 V) and a relatively low double-layer capacitance (of ca. 50 μF), resulting in low background currents comparable to those at the GCE. In contrast, the second electrode based on black raw shungite (BShE) with a carbon content of ca. 63% (*w/w*) exhibited markedly worse electrochemical properties and more than four times higher double-layer capacitance, both of which were probably due to the presence of poorly conductive impurities. The whole study has been completed with three different examples of electroanalytical applications, revealing that the first type, EShE, is a more suitable material for the preparation of electrodes and may represent a cheap alternative to commercially marketed products.

## 1. Introduction

In simple terms, rock-like shungite is metamorphosed Precambrian coal, also known as carbon–mineral composite or mineralized carbon and apparently formed by the petrification of algae through the ages. The content of carbon in shungite varies from 1 to 97%, when especially rich deposits of these fossils can be found in Karelia, on the shores of Lake Onega in north-western Russia [1,2]. Currently, there is no exact definition of shungite and, in the literature, it is variously formulated as a poorly ordered graphite with an amorphous nature (i.e., allotropic modification without a regular crystalline structure [3]), a natural form of glassy carbon [4], or even a natural metastable non-graphitized carbon with a low content of multilayered fullerene-like globules, with a diameter of 10–30 nm (<0.001% *w/w* [5]). The statement that shungite is a natural form of glassy carbon is quite misleading because the glassy carbon, consisting of two-dimensional structural elements without dangling bonds, should not be confused with amorphous carbon. In a report from 2018, Harutyunyan et al. reported the discovery (when using a high-resolution transmission electron microscopy, HRTM) that shungite consists of spatially arranged fractals of bended, curved, mono- or stacked graphene layers [6].

In 2021, it was experimentally confirmed via the use of transmission electron microscopy (TEM), Raman spectrometry and X-ray phase analysis that shungite is a rather amorphous carbon in sp2 and sp3 forms, with well-developed graphene structures of up to 200 nm [3].

Shungite can appear as a black or dark gray stone with a metallic luster (noble elite shungite), while it is always dull black after mechanical grinding. Very often, certain im-purities (mineral veins) are visible to the naked eye, namely quartz, aluminosilicates (al-bite), feldspars, carbonates, mica, pyrite, and metal oxides (Al_2_O_3_, MgO, Na_2_O, TiO_2_) [7]. Shungite rocks are usually classified, according to their carbon content, into five categories: shungite-1, with a carbon content of 90–98% (noble elite shungite); shungite-2, with a carbon content of 35–80% (black rough/raw shungite); shungite-3, with a carbon content of 20–35%; shungite-4, containing 10–20% carbon; and, finally, shungite-5, in which the carbon content is less than 10% (*w/w*) [8].

From a practical point of view, it is necessary to present a few studies that have been focused on shungite’s utilization in various branches of industry. Despite its poor pozzolanic activity, shungite gravel is already used in the construction of concrete structures as an artificial porous filler due to its long-term durability. Recently, a work was published focusing on shungite-3 containing 34.3% carbon, 56.3% SiO_2_ and 3.8% Al_2_O_3_ (all *w/w*) as an electrically conductive additive in cementitious composites to improve its mechanical properties for multifunctional applications [9].

Since shungite is characterized by its good adsorption of various organic compounds and heavy metals, as well as exhibiting antibacterial properties, it is widely used for water treatment [10]. Unfortunately, it is important to note that, at the same time, shungite can release various chemical elements into aquatic systems, including toxic heavy metals.

Another example of shungite’s practical use is the development of bifunctional oxygen electrocatalysts for the construction of metal–air batteries (MABs) and regenerative fuel cell (RFC) technologies utilizing a combination of efficient electrochemical oxygen evolution and oxygen reduction reactions [11]. Moreover, shungite was examined as a natural resource for lithium batteries and it was found that a structural modification makes it possible to exceed the energy density of common graphite [6].

Despite all other characterization studies [12,13,14], no scientific research has yet focused on a detailed study of the electrochemical properties of shungite. Therefore, there is a strong motivation to elaborate a study in which “home-made” solid-disk working electrodes are manufactured from two types (noble elite and black raw) of shungite stones and then subjected to electrochemical study, including a comparison with the most widely used and commercially available solid-disk glassy carbon electrode (GCE).

In this contribution, the electrochemical behavior of a ferro/ferricyanide redox couple and their electroanalytical performance in selected examples of practical analyses is described in detail and critically evaluated. It is demonstrated herein that the new shungite electrodes closely resemble the favorable electron-transfer kinetics of commonly used glassy carbon, whereas the kinetics parameters evaluated can be attributed to the presence of some impurities, which were identified and quantified by scanning electron microscopy and energy-dispersive X-ray spectroscopy.

## 2. Theoretical Section

This section contains a description of the basic physical relationships that were used for the individual calculations. First, cyclic voltammetric measurements in aqueous media containing the respective supporting electrolyte were performed to ensure that the only process taking place within the potential range chosen was the charging of the double layer, that is, the movement of ions on either side of an electrode/electrolyte interface. 

For ideal capacitors, a steady-state capacitive current (*i*_C_) is related to the capacitance (*C*) and to the scan rate (*ν*), as described in Equation (1) [15].

(1) $$i_{C}=\nu \;C$$

A common method for determination of the double-layer capacitance (*C*_DL_) is based on evaluation of cyclic voltammograms at various scan rates within a potential region where any redox processes do not take place. The *C*_DL_ values of the individual electrodes can be extracted from the slopes of the resulting *i*_C_ vs. *ν* linear plots. Generally, the double-layer capacitance can be used for calculation of an electrochemically active surface area (*ECSA*) if the specific capacitance of any investigated electrode material (*C*_S_) is to be determined [16], which is quite problematic in the case of heterogeneous shungite materials. For this reason, a Randles–Ševčík Equation (2) is usually used. This equation describes the effect of scan rate on the faradaic (peak) current response (*i*_p_) of a chosen redox marker (ferricyanide/ferrocyanide redox couple) [17],

(2) $$i_{p}=0.4463\mathrm{nFAC}{\left(\frac{nF\nu D}{RT}\right)}^{\frac{1}{2}}$$

where *n* is number of electrons transferred in the redox event (1 for [Fe^II^(CN)_6_]^3−^ to [Fe^III^(CN)_6_]^4−^), *F* is Faraday constant (9.64853365 × 10^4^ A s mol^−1^), *A* represents the investigated *ECSA* in unit of cm^2^, *C* is concentration of used redox marker (mol cm^−3^), *ν* is the aforementioned scan rate (V s^−1^), *D* is a diffusion coefficient (7.2 × 10^6^ cm^2^ s for ferricyanide in aqueous environment) [18], *R* is a universal gas constant (8.3144 J⋅K^−1^⋅mol^−1^) and *T* is temperature in units of K.

The same experimental data obtained from cyclic voltammetry of 1 mmol L^−1^ ferricyanide at 20–300 mV s^−1^ were used for calculating the heterogeneous rate constants (*k*^0^) in cm s^−1^ of GCE and shungite-based electrodes to compare their electrochemical reactivity (using the method of Nicholson [19]), according to Equations (3) and (4). Generally, the Nicholson method is based on kinetic parameter Ψ (dimensionless quantity) and can be used only when Δ*E*_p_ × *n* values (*Χ*) in mV fall in the range 150 ± 200 mV, where the Δ*E*_p_ is a peak separation [20].

(3) $$\Psi =k^{0}{\left[\pi Dn\nu F/\left(RT\right)\right]}^{-\frac{1}{2}}$$

(4) Ψ=(−0.6288+0.0021Χ)/(1−0.017Χ)

An exchange-current density (*j*_0_) in A m^−2^ reflects intrinsic rates of electron transfer between an analyte and the electrode; therefore, it can be considered as another important parameter for the characterization of shungite electrodes. Moreover, the exchange-current density can indicate their potential catalytic properties caused by the presence of trace amounts of metal impurities [21] and fullerenes [22]. The *j*_0_ values can be subtracted from the corresponding Tafel plots, relating the rate of an electrochemical reaction to the activation overpotential (*η*) or calculated according to Butler–Volmer Equation (5),

(5) $$j=j_{0}\;\left\{\exp\left[\frac{\alpha_{a}nF\eta }{RT}\right]-\exp\left[\frac{-\alpha_{c}nF\eta }{RT}\right]\right\}\;$$

where *j* is current density (A m^−2^), α_a_ is an anodic charge-transfer coefficient and α_c_ is a cathodic charge-transfer coefficient (both dimensionless quantities). The charge-transfer coefficient can be replaced by a barrier-symmetry factor (*β*), but applicable only to single-step reactions (see Equation (6)). The overpotential represents the potential difference between a half-reaction’s thermodynamically determined reduction potential (*E*_eq_) and the potential at which the redox event is experimentally observed (*E*), according to Equation (7).

(6) $$j=j_{0}\;\left\{\exp\left[\frac{\left(1-\beta \right)nF\eta }{RT}\right]-\exp\left(-\frac{\beta nF\eta }{RT}\right)\right\}$$

(7) $$\eta =E-E_{\mathrm{eq}}$$

In addition, the charge-transfer coefficient (α) and number of electrons (*n*) can be calculated from evaluation of peak potentials ($E_{p}^{a}\;\mathrm{and}\;E_{p}^{c}$) using the method described by Laviron [23], according to Equations (8) and (9). Laviron’s approach can be used only for cases when the peak separation (Δ*E*) is higher than 200/*n* in mV. From a practical point of view, slopes of linear parts of the corresponding trumpet plots are used into calculations. Therefore, knowledge of a standard redox potential ($E^{0}$) is not needed because it is not used in calculations.

(8) $$E_{p}^{c}=E^{0}-\frac{2.3RT}{\alpha nF}\log v$$

(9) $$E_{p}^{a}=E^{0}-\frac{2.3RT}{\left(1-\alpha \right)nF}\log v$$

## 3. Materials and Methods

### 3.1. Chemicals and Reagents

Standard solutions of heavy metals (1000 mg L^−1^ Bi^3+^, Pb^2+^, Cd^2+^ and Zn^2+^) suitable for atomic-absorption spectrometry, ≥99.0% acetaminophen, 99.9% dopamine hydrochloride, sodium dodecyl sulfate, sodium decyl sulfate, sodium octyl sulfate, cetylpyridinium chloride (CPC), polyvinyl chloride (PVC) and tetrahydrofuran (THF) from Merck (Darmstadt, Germany) were used to demonstrate electroanalytical usefulness of shungite electrodes. Sodium hydroxide, 98.5% boric acid, 85% phosphoric acid, glacial acetic acid, di-sodium hydrogen phosphate 12-hydrate, 98.5% sodium dihydrogen phosphate dihydrate and 99% sodium acetate anhydrous from Lach-Ner s.r.o. (Neratovice, Czech Republic) were chosen for preparation of Britton–Robinson buffer (BRB) of 0.1 mol L^−1^, phosphate buffer (PB) of pH 7 and acetate buffer (AcB) of pH 4.5. All these working solutions were prepared using deionized water with resistivity of 18.3 MΩ cm, which was obtained from the Millipore Milli-Q^®®^ purification unit from Merck (Darmstadt, Germany).

### 3.2. Electrode Manufacturing

**Scheme 1:**
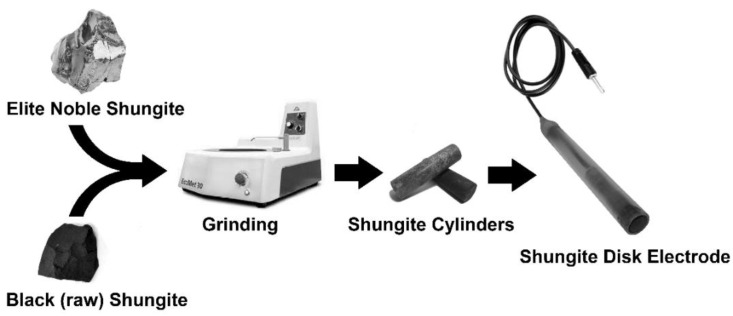
Individual steps within fabrication of disk-shungite electrodes.

Solid-disk shungite electrodes were manufactured by grinding into the shape of a cylinder (diameter of 3–9 mm and a height of 10 mm) using EcoMet 30 manual grinding and polishing machine from Hanyko Praha s.r.o. (Praha, Czech Republic) at sanding papers WS flex 18 C, self-stick, with silicon carbide of different grit levels (60–320) from Hermes Schleifmittel GmbH (Hamburg, Deutschland). The resulting cylinders were placed into cavity of plastic pipes and electrically connected using metal springs with electric cables, as demonstrated in Scheme 1.

#### Mechanical-Surface Pretreatment

Prior to each electrochemical experiment, surfaces of EShE (*d* = 8 mm) and BShE (*d* = 9 mm) were treated similarly to GCE (type 6.09395.014) with disk diameter of 3 mm, as indicated in the protocol from the manufacturer Metrohm (Prague, Czech Republic), by polishing with alumina-water suspension (Al_2_O_3_; particle size of 1.0 μm) for 30s. After subsequent rinsing of their surfaces by stream of distilled water, electrodes were ready for new electrochemical experiment. Since shungite is a material of lesser hardness than glassy carbon, it was also necessary sometimes to rinse the polishing plate to remove the resulting carbon powder. The stability of shungite materials was verified by means of repeated measurements of dopamine and acetaminophen in simultaneous analyses.

### 3.3. Apparatus

All electrochemical experiments were performed in a standard voltammetric cell containing 10 mL of the working electrolyte and incorporating the GCE, EShE or BShE, followed by Ag/AgCl/3 mol L^−1^ KCl from Metrohm as the reference and a platinum sheet from Elektrochemické detektory (Turnov, Czech Republic), acting as auxiliary electrode. The selected electrode setup was connected to a potentiostat/galvanostat Autolab (model PGSTAT101) operated through NOVA 1.11 software, both from Metrohm. Aqueous solutions of 0.1 mol L^−1^ BRB (pH 2, 7 or 12), 0.1 mol L^−1^ PB (pH 7) and 0.1 mol L^−1^ AcB (pH 4.5) served as the working electrolyte, applied in all electrochemical measurements.

Scanning electron microscopy (SEM) utilizing the backscattered-electron (BSE) and secondary-electron (SE) modes for imaging the microstructures of two different shungite stones and energy-dispersive X-ray spectroscopy (EDX) for semi-quantitative determination of encapsuled impurities were carried out using a scanning electron microscope TESCAN VEGA3 SBU with EDX probe Bruker X-Flash Detector 410-M from TESCAN s.r.o. (Brno, Czech Republic).

### 3.4. Electrochemical Experiments

Potential ranges at EShE in comparison with GCE were investigated, depending on the selected pH (2, 7 and 12) of 0.1 mol L^−1^ BRBs. For this purpose, linear-sweep voltammetry (LSV) with a potential step (*E*_step_) of 2.5 mV and a scan rate (*ν*) of 50 mV⋅s^−1^ was employed for the determination of limiting values of the anodic ($E_{\lim}^{a}$) and cathodic ($E_{\lim}^{c})$ potential.

To determine values of double-layer capacitance (*C*_DL_), cyclic voltammetry (CV) measurements of pure 0.1 mol L^−1^ BRB (pH 7) on GCE, EShE and BShE were carried out at *E*_step_ = 5.0 mV and *ν* = 0.05, 0.10, 0.25, 0.50, 1.00 and 1.50 V s^−1^. Analogical experiments with 1 mmol L^−1^ K_4_[Fe(CN)_6_] in 0.1 mol L^−1^ BRB (pH 7) at *E*_step_ = 5.0 mV and *ν* = 20, 40, 60, 80, 100, 120, 140, 160, 180, 200, 220, 240, 260, 280 and 300 mV s^−1^ were performed to determine the electrochemically active surface areas (*ECSA*), heterogeneous rate constants (*k*^0^), exchange-current densities (*j*_0_) and charge-transfer coefficients (α).

## 4. Results and Discussion

### 4.1. Microscopic Analysis of Shungite Stones

**Figure 1:**
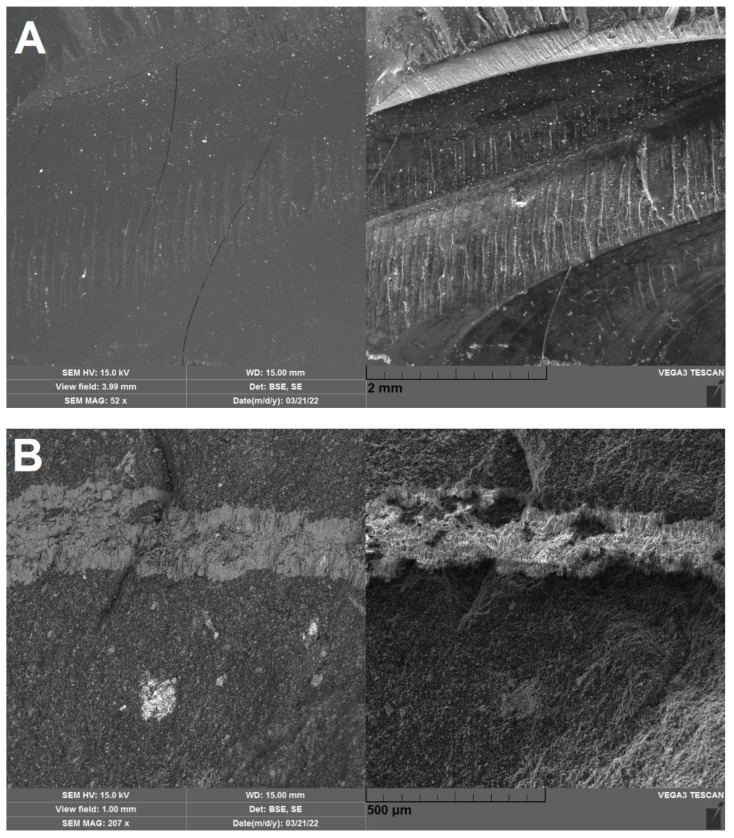
Detailed backscattered-electron (BSE) and secondary-electron (SE) images of elite shungite (**A**) and black (raw) shungite (**B**) stones with the obvious presence of a quartz vein and pyrite druses.

In general, shungites can be defined as heterogenous carbonaceous materials in which the elemental compositions are quite variable [1]. Hence, in this study, two different types of shungite stone had to be investigated using microscopic analysis to reveal their different surface topographies (see Figure 1). In contrast to the rather smooth homogeneous noble shungite, which resembles glassy carbon, black raw shungite can be considered as a fragmented (rough-surfaced) material with the admixture of many impurities (compare results from their EDX analyses in Table 1), forming veins (quartz) or druses (aluminosilicates and pyrite) of minerals. Because noble shungite is a very pure form of carbon, it should be preferred over others for the preparation of shungite-based solid electrodes applicable in electrochemical analysis. Despite this statement, the black raw shungite was subjected to further electrochemical investigation.

### 4.2. Electrochemical Characterisation of Shungite Electrodes

#### 4.2.1. Residual Currents

**Figure 2:**
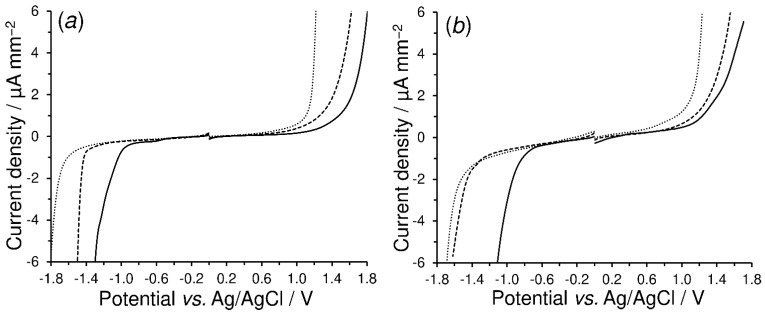
Linear-sweep voltammograms of 0.1 mol L^−1^ BRB of pH 2 (solid), 7 (dashed), 12 (dotted line) recorded on GCE (**a**) and EShE (**b**) at *E*_step_ = 2.5 mV and *ν* = 50 mV s^−1^.

In general, the potential range of the new shungite-based electrodes has a fundamental effect upon the scope of their electroanalytical utility. In comparison with the conventional GCE, the EShE does not display as a broad potential range and background currents, as shown by the linear-sweep voltammograms in Figure 2. For a still-acceptable value of background currents of about 0.5 μA mm^−2^, the anodic limit potential ($E_{\lim}^{a}$) at the GCE caused by oxygen evolution is shortened with increasing pH, namely to 1.5 V at pH 2, +1.3 V at pH 7 and +0.9 V at pH 12, whereas the cathodic limit potential ($E_{\lim}^{c}$) caused by hydrogen evolution is extended to −1.0 V at pH 2, −1.1 V at pH 7 and −1.3 V at pH 12.

On the other hand, a similarly broad cathodic range and slightly shorter anodic window were observed for shungite-based electrodes. Overall, a relatively broad potential range of ~2.1 V can be obtained for EShE in the neutral BRB (~2.4 V for GCE). From a practical point of view, both types of shungite electrode resembled rather heterogeneous carbon materials, such as carbon ink for the preparation of screen-printed electrodes or carbon-paste electrodes [24] and, therefore, they can find a wider applicability in various areas of electroanalysis [25,26].

#### 4.2.2. Double-Layer Capacitance

**Figure 3:**
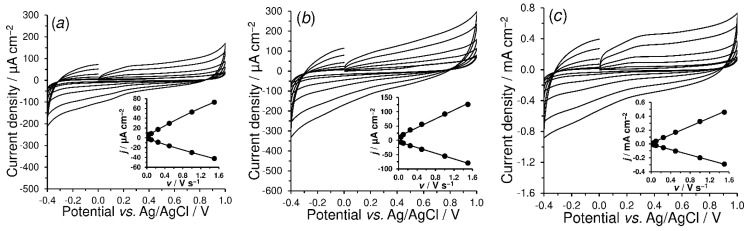
Double-layer-capacitance measurements obtained by cyclic voltammetry of 0.1 mol L^−1^ BRB (pH 7) recorded on GCE (**a**), EShE (**b**) and BShE (**c**) at *E*_step_ = 5.0 mV and *ν* = 0.05, 0.10, 0.25, 0.50, 1.00 and 1.50 V s^−1^. Inserted figures represent plots of background-current response at 0 V vs. scan rate.

As shown in Figure 3, cyclic voltammetry of pure 0.1 mol L^−1^ BRB (pH 7) from −0.4 to +1.0 V at a scan rate in the range of 0.05–1.50 V s^−1^ was used to determine the double-layer capacitance ($C_{\mathrm{DL}}$). The inserted graphs plot the linear dependences of the background current responses at +0.4 V on the applied scan rate for the GCE (*a*), EShE (*b*) and BShE (*c*). The double-layer-capacitance values of the EShE (45.7 μF cm^−2^) and BShE (192.0 μF cm^−2^) were found to be significantly higher than those of the commercially available GCE (27.6 μF cm^−2^). A significant increase in double-layer capacitance in the case of the BShE was probably caused by the low content of conductive carbon (62.3%) and a high proportion of impurities, predominantly SiO_2_ and FeS_2_.

#### 4.2.3. Electrochemical Properties of Shungite Materials

Generally, at the solid–liquid interface, electrochemical reactivity can be defined as a measure of the electron flow between a solid electrode and a substance in a solution. Therefore, it can be considered as the most important parameter used to characterize electrode materials. The relationship between the electrochemical performance against ferrocyanide and the composition of shungite electrodes is discussed in detail below.

At first, the stability of shungite-based electrode surfaces had to be examined. For this purpose, cyclic voltammetry with 50 consecutive cycles in 0.1 mol L^−1^ BRB (pH 7) containing 1 mmol L^−1^ K_4_[Fe(CN)_6_] was performed at a scan rate of 50 mV s^−1^. The negligible changes in the current responses of the redox marker used indicated the desired stability.

**Figure 4:**
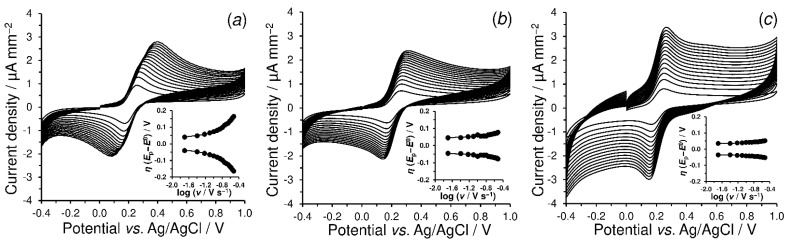
Cyclic voltammograms of 1 mmol L^−1^ K_4_[Fe(CN)_6_] in 0.1 mol L^−1^ BRB (pH 7) recorded on GCE (**a**), EShE (**b**) and BShE (**c**) at *E*_step_ = 5.0 mV and *ν* = 20, 40, 60, 80, 100, 120, 140, 160, 180, 200, 220, 240, 260, 280 and 300 mV s^−1^. Inserted figures represent the corresponding trumpet plots.

Figure 4 illustrates cyclic voltammograms at 20–300 mV s^−1^ for 1 mmol L^−1^ K_4_[Fe(CN)_6_] in 0.1 mol L^−1^ BRB (pH 7) obtained at GCE, EShE and BShE. The values of the current densities were calculated from the sizes of the corresponding geometric surfaces ($A_{\mathrm{geo}}$). 

Within this scan-rate study, it was found that anodic and cathodic peak currents ($i_{p}^{a}\;\mathrm{and}\;i_{p}^{c},$ respectively) linearly increased (R^2^ > 0.9950 for all cases) with the square root of the scan rate. Moreover, slope (*k*) values ranging from 0.438 to 0.627 for the linear dependency of the peak-current logarithm on logν were obtained, indicating a diffusion-controlled electrochemical reaction.

At first glance, the almost comparable electrochemical behavior of the ferrocyanide/ferricyanide redox couple was observed for the GCE and EShE. Due to the many-times-higher double-layer capacity of the BShE, a significant increase in the background current was confirmed. Nevertheless, it was surprising that a minimum increase in the overpotential (*η*) occurred when setting higher scan-rate values (*ν*) in the case of the shungite-based electrodes, as shown by the inset trumpet plots in Figure 4. This phenomenon can be attributed to the presence of metal impurities, especially iron-based particles (iron oxides and sulfides), which probably enhance the electron-transfer ability [27].

An advantage of BShE, compared to EShE and GCE, is the speed of electron transfer (see calculated *k*^0^ values in Table 2), which is more than doubled. This is caused by the presence of the aforementioned electrochemically active impurities (up to 20.4% *w/w* Fe), which significantly compensate the lower amount of conductive carbon (62.3–64.2% *w/w*), which is evident from the *ECSA* values in Table 2. Consequently, the comparable electrochemical properties of both types of shungite electrode with the commonly used GCE can be taken in account.

**Figure 5:**
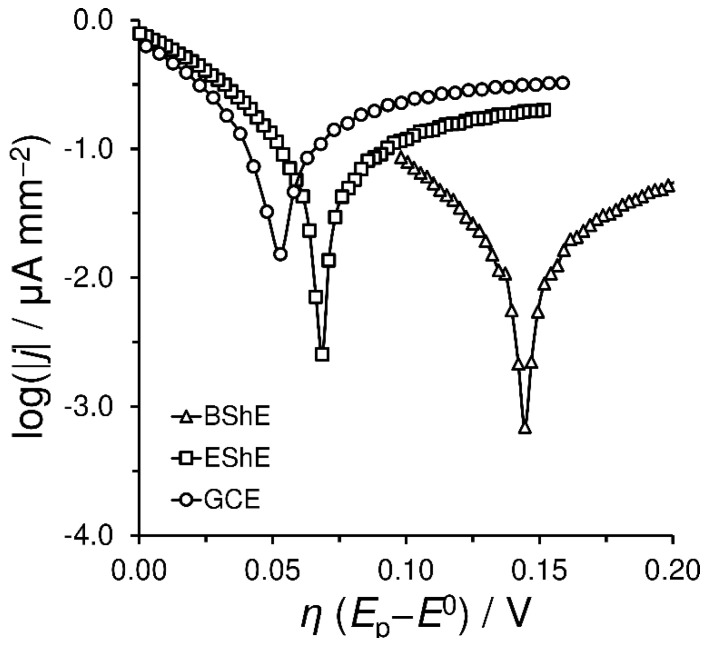
Analysis of the cathodic and anodic polarization Tafel plots obtained at BShE, EShE and GCE for 1 mmol L^−1^ K_4_[Fe(CN)_6_] in 0.1 mol L^−1^ BRB (pH 7) at a scan rate of 100 mV s^−1^.

Tafel plots for the ferrocyanide/ferricyanide redox couple obtained at the electrodes investigated are shown in Figure 5. The linear *η* vs. log *j*_c_ relationships (Tafel behaviors) were extrapolated (R^2^ > 0.9924) to yield the values of the exchange-current densities (*j*_0_) and barrier-symmetry factor (*β*_c_), which could be replaced by the charge-transfer coefficients (*α*) for the investigated single-step reaction (Fe^3+^ to Fe^2+^). As the exchange-current density reflects the spontaneous reaction rate at equilibrium potential, it can be deduced that noble elite shungite is preferable to black raw shungite, as documented by the values given in Table 2.

The obtained *α* values for the EShE and GCE that were close to a theoretical value of 0.5, indicate the symmetry of the energy barrier, i.e., anodic oxidation was not favored over cathodic reduction and vice versa. This fact was also confirmed by the calculated $\left|i_{p}^{a}/i_{p}^{c}\right|\;\mathrm{values}$ that varied from 1.018 to 0.976 for the EShE and from 0.986 to 1.090 for the GCE. By contrast, an α value of 0.45 was calculated for the BShE, indicating a preference for anodic oxidation. Furthermore, the ratio of the peak currents ranged from 1.308 to 1.102. Unlike the case of the GCE, it was typical of the shungite electrodes that the ratio of the peak currents approached the theoretical value of 1 with higher scan rates.

### 4.3. Electroanalytical Applicability

This section offers model examples demonstrating the possible applications of solid shungite electrodes in electroanalysis. Here, it is necessary to emphasize that these experiments are of an illustrative character and do not represent the fully developed electroanalytical methods. Thus, the respective working conditions and parameters are not necessarily the ultimate set-up and may require further optimization.

#### 4.3.1. Potentiometric Indication of the Surfactants

**Figure 6:**
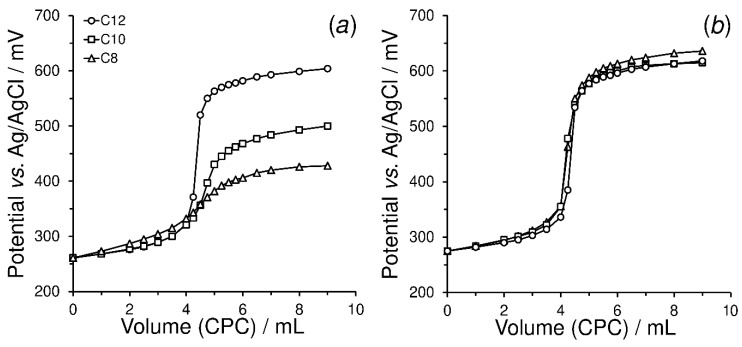
Potentiometric titration curves of three different solutions of sulfonates titrated against CPC solution (**a**). Three repetitive titrations of sodium-dodecyl-sulfate solution titrated against CPC solution (**b**).

Beyond simple potentiometric acid-base titrations, shungite has not yet been applied in the preparation of special indicators/sensors [28]. Based on some previous studies and results [29,30], the use of potentiometric titrations with coated-wire ion-selective electrodes can be taken to represent the first model example of the use of newly proposed shungite-based electrode. The indicatory electrode tested in this work was fabricated by the layering of several PVC membranes onto the BShE surface (ISE-BShE). The PVC-membrane solution was prepared by the mixing of 10 mg of graphite powder (particle size of 5 μm) from Graphite Týn, s.r.o. (Týn nad Vltavou, Czech Republic), 100 mg PVC, 200 μL 2-nitrophenyloctyl ether and 2 mL THF. This procedure was used according to the literature [31]. Typical titration curves obtained from manual potentiometric titrations of three surfactants differing in the lengths of their alkyl chains (sodium dodecyl sulfate, sodium decyl sulfate and sodium octyl sulfate, all in 0.01 mol L^−1^) with 0.01 mol L^−1^ CPC as the titrant are shown in Figure 6a. In addition, the precision of the potentiometric measurements (three representative titrations) is demonstrated in Figure 6b.

It is evident that even raw shungite can serve as a suitable electrode support for the construction of ion-selective electrodes. In the long term, the heterogeneous composition of raw shungite had no effect on the mechanical properties of the membrane. Although the potential jump significantly decreases with the length of the alkyl chain, it could be possible to analyze real samples (commercial detergents) containing surfactants of short chains, which is rather complicated to achieve with standard two-phase titration.

#### 4.3.2. Anodic Stripping Voltammetry of Heavy Metals with a Green Electrode

**Figure 7:**
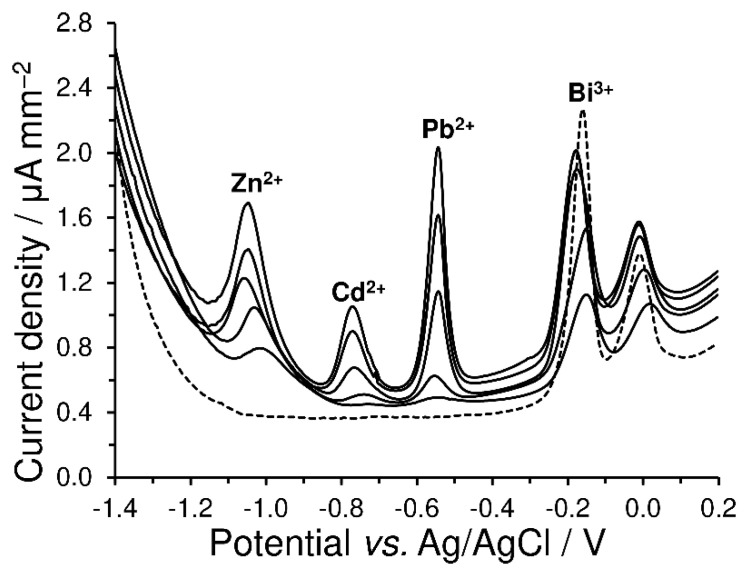
Simultaneous detection of heavy metals using SWASV at BiF-EShE in 0.1 mol L^−1^ acetate buffer (pH 4.5) containing 0 (dashed line), 10, 20, 30, 40 and 50 µg L^−1^ Zn^2+^, Cd^2+^ and Pb^2+^, always with 200 µg L^−1^ Bi^3+^, *E*_dep_ = −1.5 V for 120 s, *t*_eq_ = 10 s, *E*_step_ = 5 mV, *E*_ampl_ = 25 mV and *f* = 25 Hz, simultaneous calibration measurements.

Since noble elite shungite is a very pure carbonaceous material without the undesirable admixture of heavy metals (see Table 1), EShE was tested as the supporting electrode for a bismuth film plated in situ (BiF-EShE), which is now a very popular sensor for the trace analysis of heavy metals due to its favorable ecological profile, as well as fulfilling the criteria of so-called green chemistry [32,33]. When coupled with square-wave anodic stripping voltammetry (SWASV), this environmentally friendly electrode allows the simultaneous determination of a trio of heavy metals (zinc, cadmium and lead) within the model-calibration measurements (with 10, 20, 30, 40 and 50 μg L^−1^ of each metal ion), as documented in Figure 7.

In comparison with commonly used GCE, the use of a bismuth-film electrode plated in situ (BiF-GCE) gives rise to anodic peaks of heavy metals that are sharper and thus more distinguishable [34]. Regarding the response of bismuth itself, it has no effect on the electroanalytical determination of heavy metals, and most authors do not even show it in their resulting voltammograms. Herein, it is necessary to note that, during accumulation, special low-melting alloys of metals with bismuth (a kind of intermetallic compound [32,33]) are formed and may cause the splitting of the reoxidation peak of bismuth during the voltammetric scan or give rise to another peak near the bismuth signal. This is a usual consequence of the fact that, in the SWASV mode with a bismuth electrode prepared in situ, the concentration of Bi(III) ions should be minimally one-order higher in order to ensure the formation of proper accumulation properties in the bismuth film during the preconcentration step, which is essential in the appropriate alloying of the metal ions of interest, for which appropriate methods are proposed and optimized [33].

#### 4.3.3. Electrosensing of Acetaminophen and Dopamine

**Figure 8:**
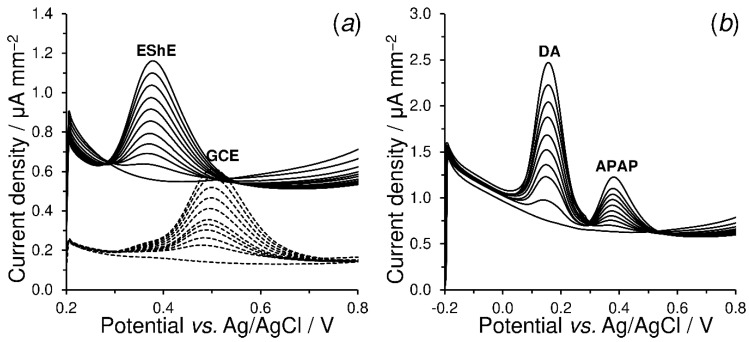

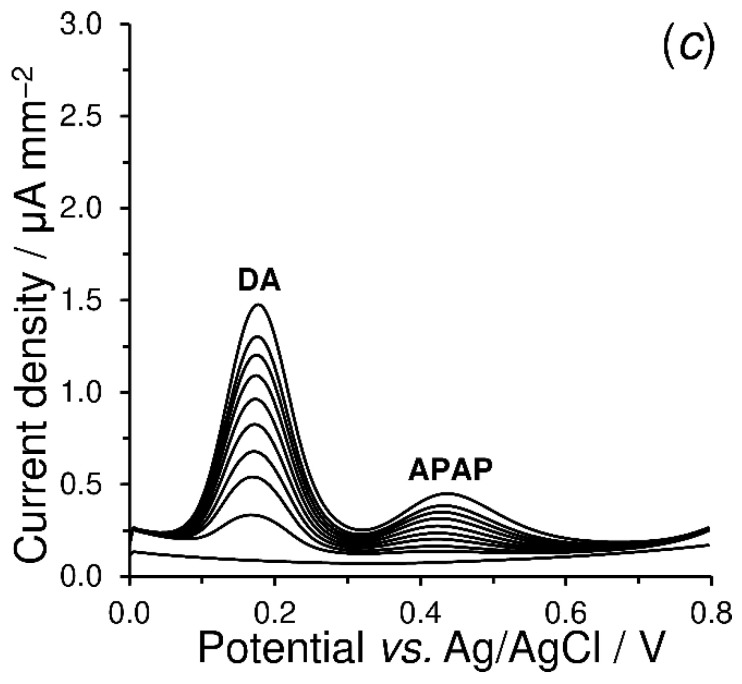
Square-wave voltammograms of 0, 10, 20, 30, 40, 50, 60, 70, 80, 90 and 100 μmol L^−1^ APAP recorded on EShE and GCE in 0.1 mol L^−1^ PB of pH 7 at *E*_step_ = 5 mV, *E*_ampl_ = 50 mV and *f* = 10 Hz (**a**). Square-wave voltammograms for the simultaneous calibration of DA (0–100 μmol L⁻^1^) and APAP (0–100 μmol L⁻^1^) recorded on EShE (**b**) and GCE (**c**) under aforementioned working conditions.

As demonstrated in Figure 8, acetaminophen (APAP), known as an antipyretic and analgetic drug, is electrochemically oxidized to *N*-acetyl-*p*-benzoquinone-imine (NAPQI) with the participation of two electrons and two protons [35] on EShE at a potential of +0.391 V, compared the higher potential of GCE, of +0.507 V. The width of the corresponding oxidation peaks was comparable for both electrodes. The obtained calibration dependences (*j* = 0.0061*c* − 0.0460 with R^2^ = 0.9989 for EShE and *j* = 0.0043*c* − 0.0048 with R^2^ = 0.9926 for GCE) show a greater sensitivity (*k*) at EShE. However, due to a higher capacitive current causing a non-negligible intercept (*q*), the method of standard addition could provide falsely lower results.

Surprisingly, individual oxidation peaks for dopamine (DA) and APAP can be distinguished using unmodified EShE, which cannot be realized on bare GCE (due to significant overlapping) [36,37]. This phenomenon is also demonstrated by the voltammetric curves in Figure 8c. The linear regression described by the equation *j* = 0.0171*c* − 0.0803 with R^2^ = 0.9976 for DA electrosensing was obtained in a phosphate buffer (PB) 0.1 mol L^−1^ with a pH of 7 at a potential step (*E*_step_) of 5 mV, potential amplitude (*E*_ampl_) of 50 mV and frequency (*f*) of 10 Hz. Replicated measurements (*n* = 5) of a mixture containing 50 µmol L^−1^ DA and 50 µmol L^−1^ APAP were performed to determine the precision, as well as to verify the stability of the electrode surface (after its renewal), when values less than ±4% RSD were found for both model analytes. The above findings indicate that the sensors derived from noble elite shungite could find their electroanalytical utilization in pharmaceutical and clinical analysis as another possible alternative materials, such as graphite sheet paper [38] and cellulose-acetate-based graphite ink [39]. Herein, it is necessary to mention that the proposed electroanalytical methods have not yet been optimized sufficiently to achieve the proper analytical performance.

## 5. Conclusions

The manufacture of completely new types of carbonaceous solid electrodes, their electrochemical properties and electroanalytical utilities were demonstrated. The resulting shungite-based electrodes were characterized with different electrochemical properties due to the variation in their elemental compositions, which is related to their place of origin (shungite deposits). The similarity between their electrochemical properties and those of conventional glassy carbon make it possible to apply noble elite shungite (type of shungite-1) electrodes in different areas of electroanalysis. However, electrodes derived from shungite stones with lower carbon contents and a stronger presence of inorganic impurities (types of shungite-2 to 5) may exhibit certain limitations due to their high double-layer capacity and slow electron transfer.

From a practical point of view, if the number of the deposits of noble elite shungite is considered, numerous sensors could be fabricated for commercial purposes. In addition, there are already shungite powders with a well-defined particle distribution (1–20 nm) that could be used for the preparation of corresponding shungite-paste electrodes with unique properties. Such configurations could widen the already existing portfolio of carbon- or graphite-powder materials that have hitherto been used and reported as applicable in carbon-paste mixtures [40]. Finally, this study should be understood as an initial step in new research, opening another field of application for shungite-based materials, in addition to their promising use as cathode catalysts [41].

## Figures and Tables

**Table 1:** Elemental composition of examined shungite materials.

Shungites	C	O	Si	Fe	S	Al	Cl	K	Mg
Noble elite	90.6–94.1	8–5.9	0.2	0.6	0.4	—	0.2	—	—
Black raw	62.3–64.2	24.7–55.2	8.8–32.3	0–20.4	0.2–7.1	0.6–1.1	—	0.5–2.1	0–0.3

Notes: Values in % (*w/w*) obtained from EDX analysis of different parts of shungite materials.

**Table 2:** Survey of calculated electrochemical parameters.

Sensor	*R*/Ω cm	*A*_geo_/cm^2^	*ECSA*/cm^2^	*C*_DL_/μF	*k*^0^/cm s^−1^	α	*j*_0_/A cm^−2^
EShE	0.3	0.502	0.311	45.7	0.0078	0.47	4.3 × 10^−6^
BShE	9.6	0.636	0.472	192.0	0.0159	0.45	8.7 × 10^−7^
GCE	4.5 × 10^−3^	0.071	0.047	27.6	0.0069	0.49	5.5 × 10^−6^

Notes: Data (excluding the first two) were obtained from cyclic voltammetry of 0 and 1 mmol L^−1^ K_4_[Fe(CN)_6_] in 0.1 mol L^−1^ BRB (pH 7) at scan rates ranging from 20 to 300 mV s^−1^.

## Data Availability

All the relevant data are only provided in the present paper.
